# Epidemiology of anti-tuberculosis drug resistance patterns and trends in tuberculosis referral hospital in Addis Ababa, Ethiopia

**DOI:** 10.1186/1756-0500-5-462

**Published:** 2012-08-28

**Authors:** Dereje Abate, Bineyam Taye, Mohammed Abseno, Sibhatu Biadgilign

**Affiliations:** 1 Addis Ababa University, College of Health Science, School of Medical Laboratory Sciences, Addis Ababa, Ethiopia; 2 St. Peter’s TB Specialized Hospital, Addis Ababa, Ethiopia; 3 Addis Ababa University, College of Health Science, School of Public health, Addis Ababa, Ethiopia; 4 Department of Epidemiology and Biostatistics, Jimma University, College of Public Health and Medical Science, Jimma, Ethiopia

**Keywords:** Epidemiology, MDR-TB, Drug resistance, Drug susceptibility, Trend

## Abstract

**Background:**

Drug-resistant TB has emerged as a major challenge facing TB prevention and control efforts. In Ethiopia, the extent/trend of drug resistance TB is not well known. The aim of this study was to determine the pattern and trend of resistance to first line anti-TB drugs among culture positive retreatment cases at St.Peter’s TB Specialized Hospital.

**Findings:**

A hospital based retrospective study was used to assess the pattern of anti-TB drug resistance among previously treated TB patients referred to St.Peter’s TB Specialized Hospital from January 2004-December 2008 Gregorian calendar(GC) for better diagnosis and treatment.

Among 376 culture positive for *M. tuberculosis* one hundred and two (27.1%) were susceptible to all of the four first line anti-TB drugs -Isoniazid (INH), Rifampicin (RIF), Ethambutol (ETB) & Streptomycin (STM). While 274 (72.9%) were resistant to at least one drug. Any resistance to STM (67.3%) was found to be the most common and the prevalence of MDR-TB was 174 (46.3%). Trend in resistance rate among re-treatment cases from 2004 to 2008 showed a significant increase for any drug as well as for INH, RIF, and MDR resistance (P <0.05 for trend).

**Conclusions:**

There has been an increasing trend in drug resistance in recent years, particularly in retreatment cases. Therefore, establishing advanced diagnostic facilities for early detection of MDR-TB and expanding second line treatment center to treat MDR-TB patients and to prevent its transmission is recommended.

## Introduction

Tuberculosis (TB) is a disease impacting public health problem worldwide that disproportionately affects peoples in resource-poor settings, particularly those in Asia and Africa. More than 90% of new TB cases and deaths occur in developing countries [1,2].

According to the 2011 WHO global TB report, Ethiopia ranks 7^th^ in the list of the world’s 22 high burden countries for TB with estimated incidence rate to be 261/100,000, mortality rate 35/100,000 and the prevalence of all forms TB is estimated to be 394/100,000 [2]. About 40-70% of HIV patients in Ethiopia are co-infected with TB [3]. Recently the country has massively engaged in early case detection, provision of adequate chemotherapy and prevention of transmission to new cases [4]. Despite such efforts, the emergence of drug resistant tuberculosis strains that cannot be cured by standard anti-tuberculosis drug regimens considered as devastating threat to TB control program [5]. Drug resistant tuberculosis commonly arises through the selection of mutated strains by inadequate chemotherapy. Resistance to at least the two major anti-tuberculosis drugs, isoniazid and rifampicin has been termed as multidrug-resistant tuberculosis (MDR-TB). Treatment of MDR-TB requires prolonged and expensive chemotherapy using second line drugs of heightened toxicity and less effective [6,7].

According to WHO Global TB report, Ethiopia is ranked as 15^th^ among 27 high burden M(X) DR-TB countries. The estimated MDR rate was (0.9%–2.8%) for new cases and (5.6%–21%) for retreatment cases [2]. Despite the availability of a national estimate, it has been noted that continues evaluation of ongoing treatment program is one of the important aspects in the assessment of TB epidemiology [8,9]. Therefore, the present study was to determine the pattern and trend of resistance to first line anti-TB drugs among culture positive retreatment cases at St. Peter’s TB Specialized Hospital in Addis Ababa, Ethiopia.

## Methods

### Research setting and context

St. Peter’s TB specialized hospital was established in June, 1961 Gregorian Calendar (GC). It is a governmental hospital under Federal Democratic Republic of Ethiopia- Ministry of Health (FMOH). The hospital provides various services especially in tuberculosis diagnosis and treatment. It serves as a referral TB hospital in Addis Ababa, Ethiopia and has a vision to become Center of Excellence for diagnosis and treatment of TB in East Africa.

### Study design and participants

A Hospital based retrospective study was conducted in Addis Ababa from February 2010 to March 2010 GC to assess the patterns of resistance to anti-TB drugs among previously treated TB patients referred to St. Peter’s TB Specialized Hospital. Participants for this study were all previously treated TB cases from January 2004 – December 2008 GC. The study subjects already received the standard first line anti-TB treatment for more than one month but still had positive smears and had to be started on a retreatment regimen (2 S (ERHZ)/1(ERHZ)/5 E3 (RH)3. The hospital provides for those patients refereed to the hospital across the country as the hospital serve as a referral point for tuberculosis management for the country.

### Drug susceptibility test

The drug susceptibility tests were done at Ethiopian Health and Nutrition Research Institute (EHNRI) TB Laboratory. The laboratory used Löwenstein-Jensen (L-J) culture medium on which the specimen was inoculated after decontamination with sodium hydroxide (4%). Identification of isolates was based on the niacin production test, the nitrate reduction test the para-nitrobenzoic (PNB) acid (500 mg/l) test, and the thiophene-2-carboxylic acid hydrazide (TCH) (2mg/l) resistance test. Mycobacteriums other than M. tuberculosis complex were excluded from the analysis [10].

Drug susceptibility tests were performed using the simplified variant of the indirect proportion method on L-J medium. The proportion method (Canetti modified) determines the percentage of growth (number of colonies) of defined inoculums on a drug-free control medium versus growth on culture media containing the critical concentration of an anti- tuberculosis drug. The critical drug concentration, as well as the critical proportion of resistant colonies were been evaluated from clinical data. Resistance was expressed as the percentage of colonies that grew on recommended critical concentrations of the drugs tested (i.e. 0.2 mg/l for isoniazid (INH), 2 mg/l for ethambutol (EMB), and 4 mg/l for streptomycin (STM) and 40 mg/l for rifampicin (RIF). The criterion used for drug resistance was growth of 1% or more of the bacterial population on media containing the critical concentration of each drug. The results of the tests were recorded on standardized forms [10].

### Data collection procedures

Laboratory based retrospective study was done among TB patients who had retreated for first line anti-tuberculosis drugs. Participant demographic variable, drug susceptibility test results of *Mycobacterium tuberculosis* (MTB) were carefully collected from the laboratory registration book using a standardized check list. The overall activities of data collection were monitored by the principal investigator and there was strict supervision during data collection. Only those patients with full information recorded in the registration book were included in the study. The extracted variables were age, sex, year of treatment, smear sample, drug resistance. Risk factors for drug resistance could not be evaluated as there was insufficient data in the charts.

### Operational definitions

Resistance in new cases (primary) was defined as in vitro resistance in patients who did not have a history of anti-TB treatment, while retreatment resistance (secondary) was defined as in vitro resistance in patients previously treated with any anti-TB medication. The definition of MDR-TB cases recommended by WHO and IUATLD is the pattern of drug resistance to at least INH and RIF. Resistance to more than one agents except these agents was referred to as poly drug resistance TB (PDR-TB) [11].

### Data management and analysis

Data collected in this study were used to ascertain the percentage of drug resistance in each year, to any drug, each drug either alone or in combination with other drugs. The percentage of resistant isolates from patients was calculated by dividing the number of resistant isolates in each of the categories by the total number of isolates tested for that drug or combination of drugs. We evaluated temporal trends in resistance to each drug alone or in combination with other drugs, as well as trends in multidrug resistance. The chi square test for trend was used to assess percentage resistance by year of report at the 5% critical value. Data analysis was conducted using SPSS Version 16.0 software (SPSS INC, Chicago, IL, USA).

### Ethical consideration

Ethical clearance for the conduct of this study was obtained from St. Peter’s TB Specialized Hospital Ethical Review Committee and Institutional Ethical Review Committee of Addis Ababa University, Faculty of Medicine; School of Medical Laboratory Science. Names or identification number of study participants were not included from the data collection template.

## Findings

A total of 376 distinct cultures positive retreated cases (previously treated with first line anti- TB drugs for at least one month) cases were identified from January 2004 to December 2008 at St. Peter’s TB Specialized Hospital Laboratory registration book. Of these 235 (62.5%) were male and the age range of the study population was 9 -74years (Mean age, 31.9), median age of 28 (9–76). Of these 87.5% was in age range of 15 – 49. The study population was 69, 83, 86, 74 and 64 across per year, respectively (Table 1).

**Table 1:** Socio-demographic characteristics of the study participants, St. Peter’s TB Specialized Hospital, 2011

**Characteristics**	**Frequency**	**Percentage (%)**
**Sex**		
Male	235	62.5
Female	141	37.5
Total	376	100
**Age**		
<15 Years	7	1.8
15-49 Years	329	86.8
>49 Years	43	11.4
Total	376	100
**Years**		
2004	69	18.2
2005	83	21.9
2006	86	22.7
2007	74	19.5
2008	64	16.8
Total	376	100

**Figure 1:**
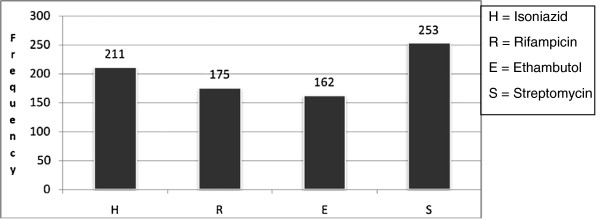
**Frequency of First line Tuberculosis Drug resistance in St. Peter's TB Specialized Hospital**, **2011.**

Among 376 culture positive for M. tuberculosis one hundred and two (27.1%) were susceptible to all of the four first line anti-TB drugs -Isoniazid, Rifampicin, Ethambutol & Streptomycin, and 274 (72.9%) were resistance to at least one drug. Resistance to any STM (67.3%) was found to be the most common followed by (56.1%) INH, (46.1%) RIF and (43.5%) ETB (Figure 1).

Seventy one (18.9%) were resistant to only a single drug while more than one drug resistance includes both poly resistance and MDR-TB. so that it should be 203 (203/376=54%) were resistant to two or more drugs. Among mono resistance, Streptomycin 56 (78.8%) was found to be highest proportion, followed by isoniazid. However, our result showed no mono resistant strain for Rifampicin.

Poly resistance was reported in 29 (7.7%) of the cases. Among these, the highest proportion was 15(53.6%) Isoniazid + Streptomycin Combination. In this study the prevalence of MDR-TB (defined as the resistance to at least INH and RIF) 174 (46.3%) was found to be high (Table 2). Among these, 103 (59.2%) were males and 154 (90.8%) were between age range of 15 – 49 years. Out of MDR-TB cases 140 (80.5%) cases were resistant for all first line anti-TB drugs.

**Table 2:** Pattern of drug resistance among 376 cultures positive retreatment cases, St. Peter’s TB specialized Hospital, 2011

**Pattern of drug resistance**	**Frequency**	**Percentage (%)**
**Any drug resistance**	274	72.9
INH^1^	211	56.1
RIF^2^	175	46.5
ETB^3^	162	43.1
STM^4^	253	67.3
**Single drug**	71	18.9
Mono INH Resistant	11	2.9
Mono RIF Resistant	0	0
Mono ETB Resistant	4	1.1
Mono STM Resistant	56	14.9
**Two drug**		
INH + RIF	4	1.1
INH + ETB	2	0.53
INH + STM	15	4.0
RIF + ETB	0	0
RIF + STM	0	0
ETB + STM	0	0
**Three or more drug**		
INH + RIF + ETB	2	0.53
INH + RIF + STM	28	7.5
INH + ETB + STM	12	3.2
RIF + ETB + STM	0	0
INH + RIF + ETB + STM	140	37.2
MDR^5^	174	46

^1^INH = Isoniazid, ^2^RIF = Rifampicin, ^3^ETB,= Ethabutol, ^4^STM, = Streptomycin, ^5^MDR = Multi drug resistance (resistance at least INH and RIF).

**Figure 2:**
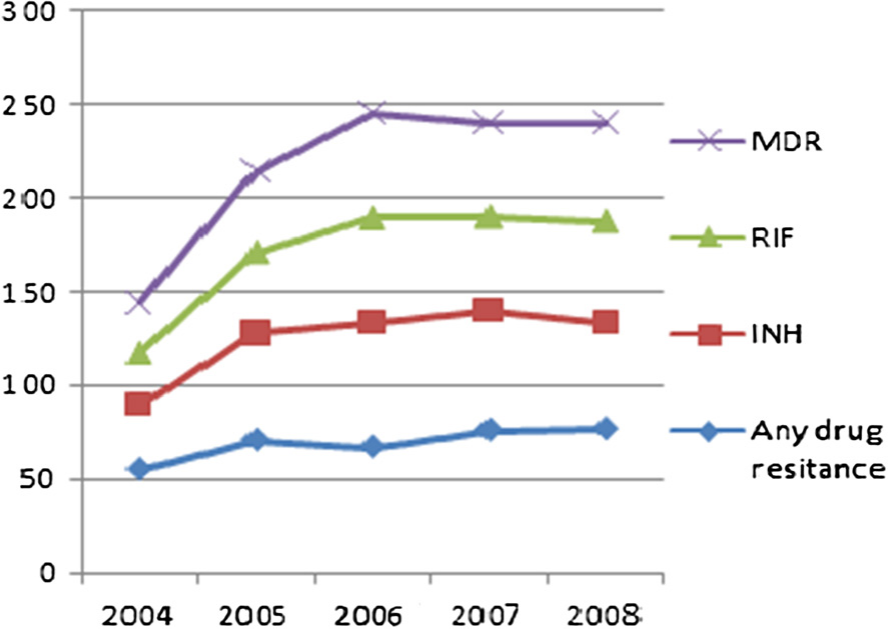
Trend in drug resistance to anti-TB drugs in re- treatment cases, St. Peter’s TB Specialized Hospital, 2011.

Any drug resistance as well as MDR-TB was not statistically significantly associated with any age group. However, there was statistically significant association between any drug resistances among gender, specifically male patients (P < 0.05) (Table 3). Trends in resistance rate among re-treatment cases from 2004 to 2008 showed a statistically significant increase for any drug as well as for INH, RIF, and MDR resistance (P <0.05 for trend) (Figure 2).

**Table 3:** Characteristics of culture positive retreatment cases according drug resistance pattern, St. Peter’s TB Specialized Hospital, 2011

**Variable**	**N (%)**	**Any drug resistance n (%)**	**p-value**	**MDR-TB n, (%)**	**P-value**
Sex					
Male	235 (62.5)	154 (59)	0.03	103(59.2)	0.220
Female	141 (37.5)	107 (41)		71 (40.8)	
Age(yr)					
<15	7 (1.9)	5 (1.9)	0.990	4 (2.3)	
15–49	330 (87.8)	235(90)	0.395	158 (90.8)	0.689
>49	39 (10.4)	21 (8)		12 (6.9)	0.333

## Discussion

In the present study, 102 (27.1%) of the cases were susceptible to all of the four first line anti-TB drugs and 274 (72.9%) were resistant to at least one or more anti-TB drugs indicating higher prevalence of drug resistant TB for first line anti-TB drugs than studies conducted in Ethiopia with overall resistance to any anti-TB drugs ranges from 14% -27% [12-16]. The higher magnitude of resistance to any anti-TB drugs in the present study could be explained the difference in the characteristics study subjects (retreatment cases) which is more likely to have resistance and majority of retreatment cases in the country referred to the study hospital.

Mono resistance to streptomycin (14.9%) was found to be the highest proportion among first line anti-TB drugs which is consistent with WHO global surveillance report [5] as well as in other studies conducted in Denmark, Turkey, India and Arsi zone of Ethiopia [17-20]. In contrast a 15-year surveillance in Saudi Arabia showed, resistance to INH and EMB was more frequent than to other first-line drugs [21].

MDR-TB in retreatment patients varies from 30% to 80% in different regions [22] and in Ethiopia, MDR –TB prevalence in 2008 was (0.9%–2.8%) for new cases and (5.6%–21%) for retreatment cases [2].

In this study, MDR-TB was found in 46.3% of retreatment; this shows a higher percentage of MDR-TB in Ethiopia, particularly in retreatment cases, compared to WHO report [2]. Surprisingly, our study noted that MDR-TB prevalence is significantly higher in male than female patients which are inconsistent with a report by *O’Donnell MR et al.* where female have more risk than male patients [23]. The latter study explained as gender differences in drug-resistant TB in areas of HIV endemicity and low prevalence suggest a possible effect of the HIV/AIDS epidemic on prevalence of drug-resistant TB in women [24]. Despite such difference, male or female TB patients could have different levels of risks for drug resistance due to differences in access to health-care services or exposure to other risk factors. Discovering gender disparities associated with the risks of MDR-TB could provide insight into the development of targeted measures and improve access to health care and reduce the risk of acquiring drug-resistance.

The association between age and the risk of MDR-TB is not well established in the literatures as different studies use different cut-off points for age groups. MDR-TB patients were more likely to be younger than 65 years [24,25]. A systematic review analysis in Europe revealed the pooled risk of MDR-TB for people younger than 45 was higher than that among older patients (OR: 1.52, 95% CI: 1.13-2.03) [24]. In this study, we did not found a statistical significant association between patient’s age and frequency of MDR-TB which was in contrast with a report by Shao et al. indicating MDR –TB was much higher in young adulthoods and peaked at 35–44 years old [26].

The trend in drug resistance against all first-line drugs shows a significant increase. This remarkable percentage of drug resistance among retreatment patients supports the idea of inefficiency in TB control programs and irregular/improper anti-TB drug use in recent years, which have led to accumulation and multiplication of resistant strains. Notably, resistance to the other corner stone of anti-TB therapy, RIF mono resistance, was not observed in current study, but there is a significant increase in proportion of INH resistance which may be considered as an alarming sign and indicates further transmission of resistant strains in the community.

The study has its own limitations. There is a potential bias in estimation of drug resistance in previously treated cases and the data were taken from the hospital, it may be subjected to selection bias, and it was not possible to get other relevant variables such as co-morbidity, HIV status. In resource poor settings where the quality of reports are often questionable and the retrospective data collected primarily for reporting may not be robust [27].

## Conclusions

In conclusion, the high prevalence of drug resistance in this study area may limit the success of TB control program. Increased trend in resistance to anti-TB drug among re-treatment cases were noted. So this signifies that establishing advanced diagnostic facilities for early detection of MDR-TB. Monitoring of drug-resistance should be enhanced by periodic surveys to assess trends and take correct actions when necessary. Prevention and control of drug-resistant TB should be emphasized by the revised DOTS program through prompt case detection, routine and quality-assured DST for those patients at high risk of resistance, proper administration of anti-TB drugs according to the recommended national guidelines and strengthening national DOTS program, expanding second line treatment used for treating the existing cases and help to protect its transmission, This enables to appropriately manage TB patients and to lower the rate of MDR-TB among retreatment cases. In addition further studies and continued surveillance of the resistance pattern of *M. tuberculosis* is needed to further delineate the risk factors and to formulate the plans for the future management of tuberculosis in high MDR-TB settings.

## Competing interest

All authors declare that they have no conflict of interest associated with the publication of this manuscript.

## Authors’ contributions

DA conceived and designed the study and collected data in the field, performed analysis, Interpretation of data, and draft the manuscript. BT assisted with the design, performed analysis, interpretation of data and the critical review of the manuscript. MA participated in design and performed analysis, interpretation of data, helped in drafting the manuscript and critically reviewed the manuscript. SB participated in interpretation of data, helped in drafting the manuscript and critically reviewed the manuscript. All authors read and approved the final version of the manuscript. All authors participated in critical appraisal and revision of the manuscript.
